# Reframing Optimal Control Problems for Infectious Disease Management in Low-Income Countries

**DOI:** 10.1007/s11538-023-01137-4

**Published:** 2023-03-12

**Authors:** Morganne Igoe, Renato Casagrandi, Marino Gatto, Christopher M. Hoover, Lorenzo Mari, Calistus N. Ngonghala, Justin V. Remais, James N. Sanchirico, Susanne H. Sokolow, Suzanne Lenhart, Giulio de Leo

**Affiliations:** 1 Department of Mathematics, University of Tennessee, Knoxville, TN USA; 2 Dipartimento di Elettronica, Informazione e Bioingegneria, Politecnico di Milano, Milano, Italy; 3 Division of Environmental Health Sciences, University of California, Berkeley, Berkeley, CA USA; 4 Department of Mathematics, University of Florida, Gainesville, FL USA; 5 Environmental Science and Policy, University of California, Davis, Davis, CA USA; 6 Stanford Program for Diseases Ecology, Health and the Environment, Stanford University, Pacific Grove, CA USA; 7 Department of Earth System Science and Department of Oceans, Hopkins Marine Station, Stanford Doerr School of Sustainability, Stanford University, Pacific Grove, CA USA

**Keywords:** Optimal control of differential equations, Epidemiological management goals

## Abstract

Optimal control theory can be a useful tool to identify the best strategies for the management of infectious diseases. In most of the applications to disease control with ordinary differential equations, the objective functional to be optimized is formulated in monetary terms as the sum of intervention costs and the cost associated with the burden of disease. We present alternate formulations that express epidemiological outcomes via health metrics and reframe the problem to include features such as budget constraints and epidemiological targets. These alternate formulations are illustrated with a compartmental cholera model. The alternate formulations permit us to better explore the sensitivity of the optimal control solutions to changes in available budget or the desired epidemiological target. We also discuss some limitations of comprehensive cost assessment in epidemiology.

## Introduction

Due to systematic pervasive nonlinearities and heterogeneities underlying infectious disease transmission at the population level, epidemiological modeling is a key tool for exploring the potential impact of candidate intervention strategies (Bubar et al. 2021). Questions surrounding the best timing, target populations, and treatment effort to limit the transmission of communicable diseases—whether through drug treatment, vaccines, or non-pharmaceutical interventions—have been central in epidemiological modeling since the publication of the first malaria models by Ross and MacDonald more than a century ago (Smith et al. 2012). Optimal control techniques can be used to address such questions (Sharomi and Malik 2017).

Identification of optimal control strategies for managing diseases has long been pursued informally. In the absence of health interventions or natural immunity, an infectious disease follows its epidemic curve and may cause substantial morbidity and mortality (Bjørnstad et al. 2020), but by investing resources in control and treatment, it is possible to reduce the burden of disease (Keeling and Rohani 2008), even as the decision space in selecting certain strategies is large with uncertain impacts. Larger control and efforts can lower disease burden while increasing costs of control, yet these relationships are often nonlinear, with decreasing returns on investment—i.e., health benefits per dollar invested—at high control effort (Ozawa et al. 2021). Accordingly, extreme control efforts may be infeasible (possibly due to vaccine hesitancy Sallam et al. 2022) or prohibitively expensive (Ozawa et al. 2021). In addition, extreme control effort can be associated with undesirable outcomes, such as intervention fatigue (Rypdal et al. 2020). Intermediate levels of control may help manage costs of disease control when accounting for both the intervention costs (which depend nonlinearly on the control effort) and the cost associated with the burden of disease, but establishing the specific effort levels across strategies remain challenging.

Optimal control (OC) theory has proved to be a particularly powerful mathematical tool to identify the best strategies for the control of infectious diseases. In contrast to some optimization approaches that assume control variables to be constant in time, the admissible control variables in OC (e.g., vaccination rate, drug treatment) can vary in time, allowing dynamic minimization of both the cost of disease burden and the costs of intervention.

In the last two decades, the number of published papers on OC applications to the control of infectious diseases of public health or veterinary importance has increased exponentially. In the majority of these papers, disease burden is typically represented by the integral of *I*(*t*), the number of infectious individuals at time *t*, and the objective functional to be optimized is generally formulated in monetary terms as the sum of intervention costs plus the cost associated with the burden of disease, assuming that both can be estimated monetarily with a reasonable degree of precision (Asano et al. 2008; Neilan et al. 2010; Agusto et al. 2020; Jung et al. 2009; Lee et al. 2020b). This formulation has been very useful to formalize the trade-off between the goal to minimize disease burden and, at the same time, the intervention costs. Yet, as discussed below, accounting for disease burden and loss of human lives in monetary terms is fraught with uncertainties and generally considered not ethically and socially acceptable from a public health perspective (Baltussen 2006; Brock and Wikler 2006; Jiang et al. 2020; Morrow and Byrant 1995; Rutstein et al. 2017).

Minimization of the cost of disease burden represented by the integral of *I*(*t*) combined with minimizing the cost of control action has been common, but there are notable exceptions. Examples of alternative formulations of the optimal control problem that do not require monetization of health outcomes are available in the literature. Some solve the OC problem under budget constraints and optimize disease burden. Others change the representation of the goal in terms of other disease outcomes. For instance, Hansen and Day optimized the total number of cases in an ${\textit{SIR}}$ model with control of vaccination, subject to resource constraints (Hansen and Day 2011). Also Bolzoni and collaborators used OC theory to minimize the time to drive the current prevalence level to a desired threshold under resource limitation (Bolzoni et al. 2017, 2019). Using vaccination as a control in a ${\textit{SIRV}}$ model, Laguzet and Turinici minimized infections under the public health budget constraints (Laguzet and Turinici 2015). Campo-Duarte et al. (2018) used optimal control techniques for a trade-off between achieving a fixed desired level of mosquitoes not infected with ${\textit{Wolbachia}}$ in minimum time and minimizing the cost of control interventions. Yet, until recently, these examples have been rare and most of the OC published papers keep formulating OC problems with the traditional approach, i.e., minimization of the combined monetary costs of control measures and disease burden, without discussing the practical challenges of estimating the monetary cost of health outcomes.

Recently COVID-19 modeling research has pursued approaches aligned with those proposed herein. With a goal of minimizing the costs of implementing transmission rate control (social distancing measures), Miclo et al. (2022) included an upper on the number of infecteds in an ${\textit{SI}}$ model; this upper bound was interpreted as intensive care unit constraint (with infinite time horizon). Also using an ${\textit{SI}}$ system, Bliman and Duprez (2021) used social distancing controls on finite time intervals (using one dimensional optimization) to minimize the final epidemic size. Using optimization of finite duration time intervals for reduction of the transmission rates, Morris et al. (2021) minimized epidemic peak size and demonstrated optimal and nearly optimal solutions for an ${\textit{SIR}}$ model. Angulo et al. (2021) studied a control problem to steer an *SI* model trajectory to a target set with a low *I* level in minimum time (using transmission reduction control). In summary, some of these papers kept the control level low (to minimize the economic and social costs of limiting economic activity) while achieving the epidemiological constraints (upper bound on number of infecteds) (Bliman and Duprez 2021; Morris et al. 2021). Another included choices of intervals to implement the control at its maximum level while imposing an upper bound on the number of infecteds (Miclo et al. 2022). Matrajt et al. (2020) used discrete optimization to find optimal vaccination levels (taking on discrete values) for different age groups in a deterministic COVID-19 system. These examples show that, when epidemiological modeling is guided by a pressing public health problem and is used to inform the decision-making process, it is crucial to formulate the OC problem to closely reflect the specific goals and constraints.

In this paper, after briefly reviewing the practical and ethical challenges of assessing the monetary value of disease burden and of human life, we propose a simple but general framework to reformulate optimization problems to leverage the strengths of optimal control theory and, at the same time, to explicitly account for real-world features such as budget constraints and epidemiological goals that public health authorities struggle with in their daily work. We demonstrate the new framework and discuss the pros and cons of alternative formulations of OC problems using a simple but general cholera model.

### The Challenges of Estimating the Monetary Value for Health Outcomes

In industrialized countries with developed public health, welfare and demographic reporting systems, both the private and public sectors (i.e., health insurance and health departments/agencies) routinely assess the economic impact associated with diseases, accounting for the cost of over-the-counter and prescribed drugs, medical visits, hospital admissions, special treatments, loss of productivity, etc. This systematic monitoring and reporting effort provides a reasonable foundation to comprehensively estimate the monetary costs of disease outbreak and a range of public health interventions, which can be used to explore alternative control scenarios within an OC theory framework. However, this approach is not exempt from limitations. First, monetization of human health is ethically debatable, especially in the case of fatal diseases and when diseases disproportionately affect a specific segment of the population, such as underrepresented minorities, essential workers, or the homeless population (Scovronick et al. 2020).

Second, monetization of human health and/or loss of productivity becomes particularly problematic in low-income countries, plagued by a wide range of Neglected Tropical Diseases (including several infectious diseases, such as schistosomiasis, Chagas disease, dengue fever, guinea worm disease, echinococcosis, human African sleeping sickness, and leishmaniasis) and other diseases of poverty (such as malaria, tuberculosis, cholera and HIV) affecting altogether more than 2.5 billion people worldwide (Ntuli 2020). For these countries, where a non-marginal fraction of the population is often engaged in a rural, cashless economy, using the same monetary currency to contrast the costs of public health interventions with the burden of disease—which may include also reduced capacity to perform physically engaging activities in subsistence agriculture, or the long-term impact of chronic infections—is highly challenging and debatable. Some studies have explicitly recognized these challenges and analyzed OC problems as a function of loosely defined “relative costs of disease burden” with respect to the cost of intervention (see for instance, Bolzoni et al. 2014). Yet, the problem of integrating economic and public health metrics and indicators measured on vastly different units in a single monetary scale remains unsolved. Cost-effectiveness analysis partially circumvents these ethical shortcoming and practical challenges, as it abstains from measuring the monetary value of health outcomes but does not leverage the power of OC theory to identify time-varying control strategies superior to time constant strategies. While assessing the monetary value of human health is fraught with uncertainties, even the estimation of intervention costs and its translation into model parameters present formidable theoretical and practical challenges that are rarely recognized and accounted for in the published literature on OC applications to infectious disease management.

### Reframing Optimal Control Problems

Public health authorities do not operate in a vacuum, but need to plan health interventions under budget constraints and/or to achieve specific health outcomes, such as to avoid overwhelming the health system by limiting the number of people requiring hospitalization or use of the intensive care units (Miller et al. 2020). Accordingly, OC problems can be reformulated to fully embrace this more practical approach to the control of communicable diseases. Specifically, we argue that OC problems can be restated in one of the two following broad and more meaningful ways, namely:

- Alternative 1: minimize disease burden (the goal), measured in the most suitable, non-monetary units for the specific disease system under study for a given budget (the constraint) (Murray et al. 2012);
- Alternative 2: minimize the monetary cost of interventions (the goal) to achieve a specific epidemiological outcome, set as a requirement of the control strategies to be admissible (the constraint).

The first alternative recognizes that budget constraints are a significant factor in choosing control strategies in low-income countries. In the second alternative, the emphasis is on achieving a desired epidemiological target in a cost effective way. In the 2021 United Nations Political Declaration on HIV and AIDS, for example, there is a goal of reducing annual new HIV infections to under 370,000 and annual AIDS-related deaths to under 250,000 by 2025 (Assembly 2021). Optimal control policies that do not exceed regional hospital capacity and the number of available ICU beds at the peak of a COVID-19 outbreak are examples of the second alternative formulation (Miller et al. 2020; Miclo et al. 2022). Other examples include minimization of the time to a desired reduction in disease prevalence or incidence (Angulo et al. 2021; Bolzoni et al. 2017, 2019).

Here below, we present the application of these two alternative frameworks for the formulation of OC problems by using the control of a cholera outbreak as a reference example. Specifically, we use a classic mechanistic, compartmental model for cholera, based on set of ordinary differential equations, that was kept deliberately simple for demonstration purposes to show that it is not only straightforward to reframe optimal control problems in these two alternative ways, but also more informative. In fact, we show that under the new frame (i) analyses can be easily performed to assess how the outcome changes with respect to, say, increasing budget (Alternative 1) or more ambitious epidemiological goals (Alternative 2) and (ii) the results of these analyses can be presented in terms of marginal utilities, for example, additional lives saved per extra dollar invested with respect to baseline.

The rest of the paper is structured as follows. After describing the cholera model with vaccination rate as the control variable, we first define the classic problem as the minimization of the sum of the cumulative cost of disease, estimated in monetary terms, and of the cost of intervention over a given time period, *assuming the conversion factor from number of infected cases to dollars is known*. This classical approach is referred to in this paper as the “combined” OC problem.

Then, we reformulate the OC problem in the two alternative ways described above. We show that, if the conversion coefficient to assess the monetary value of disease cost can be estimated with reasonable precision, the outcome of the alternative OC formulations can be mapped back to the combined OC problem with no loss of information.

Lastly, we show that the two alternative formulations permit us to better explore the sensitivity of the OC solutions to changes in available budget or the desired epidemiological target. We conclude the paper by discussing our results and presenting some limitations of comprehensive cost assessment in epidemiology.

## Cholera Model

We illustrate our points using a basic model of cholera over a short time period. Cholera is a waterborne diarrhoeal illness caused by infection of the intestine with the bacterium *Vibrio cholerae* with both direct and indirect modes of infection and transmission, primarily through contaminated food and water supplies. The disease causes rapid dehydration and electrolyte imbalances. There are an estimated 3–5 million cholera cases and 100,000–120,000 deaths due to cholera every year (Ali et al. 2015; Legros 2018). The recent outbreak in Haiti caused over 10,000 deaths and over 820,000 cases (Zarocostas 2017; Lee et al. 2020a). The disease has a short incubation period, between two hours and five days, affecting all ages. It is known that cholera persists in an aquatic reservoir and can exist in non-culturable, but viable, state for months to over a year (Melbourne 2011). There are two types of safe and effective oral cholera vaccines, Dukoral and Shanchol (Sèvére et al. 2016).

Tien and Earn (2010) extended the classic Susceptible (*S*), Infected (*I*) and Recovered (*R*) SIR model by Codeco (2001) to include a concentration of vibrios (*W*) in the water compartment. This allows for both direct (fecal-oral) and indirect (from water-borne pathogen) transmissions pathways for cholera at per capita rates $\beta _I$ and $\beta _W$ [$\text {day}^{-1} \text {individuals}^{-1}$], respectively. For comparisons of several models applied to outbreaks in Haiti and with vaccination scenarios, see the work of Lee et al. (2020a). Our basic ${\textit{SIRW}}$ model with four state variables is described by the following differential equations:

$$\begin{aligned} S'&= \mu N -\beta _{\textit{ISI}}-\beta _{\textit{WSW}} - \mu S -v(t)S\\ I'&= \beta _W SW + \beta _I SI - \gamma I - \mu I -\delta I\\ R'&= \gamma I - \mu R +v(t) S\\ W'&= \xi ( I - W ) \end{aligned}$$

where $N = S+I+R$ represents the human population, $\mu$ is the natural mortality and birth rates of humans [$\text {day}^{-1}$], $\gamma$ is the recovery rate from infected to recovered and resistant class [$\text {day}^{-1}$], and $\delta$ is the death rate due to the disease [$\text {day}^{-1}$]. Note that the compartment *W* has been scaled to be in the same units as the *I* compartment (individuals). Therefore, $\xi$ is a parameter representing both the per capita shedding rate of viable infectious propagules in the water by an infected individual *I* and the decay rate of the pathogen in the environment [$\text {day}^{-1}$]; see more details in Tien and Earn (2010) and Kelly et al. (2016). The sum of the terms $\beta _I I$ and $\beta _W W$ are the force of infection, representing the rate at which susceptible individuals become infected either via contaminated fecal-oral transmission or the ingestion of contaminated water. The parameters and their units are described in Table .

As in Kelly et al. (2016), we assumed that susceptible individuals *S* are vaccinated and become resistant *R* at a rate *v*(*t*), with *v*(*t*) combining efficacy and rate of vaccine distribution [$\text {day}^{-1}$]. The time varying rate *v*(*t*) of vaccination is then our control variable. The basic reproduction number for this model in the absence of the control *v*(*t*) is

$$\begin{aligned} R_0 = \frac{(\beta _I+\beta _W)S^*}{\gamma + \delta + \mu }, \end{aligned}$$

where $S^*$ is the total susceptible population in the absence of disease.

**Table 1:** Parameter descriptions with units

Parameters	Description	Units
$\mu$	Natural birth/death rate	$\text {day}^{-1}$
$\beta _I$	Direct transmission rate (*P*–*P*)	$\text {day}^{-1} \text {individuals}^{-1}$
$\beta _W$	Indirect transmission rate (*W*–*P*)	$\text {day}^{-1} \text {individuals}^{-1}$
$\gamma$	Transition rate from *I* to *R*	$\text {day}^{-1}$
$\xi$	Contamination/pathogen decay rate in water	$\text {day}^{-1}$
$\delta$	Disease-induced death rate	$\text {day}^{-1}$

## Optimal Control Formulations

We begin with a discussion of formulations of objective functionals. The combined objective functional is the sum of the cumulative costs associated with the disease $J_b(v)$ and the cost of intervention $J_c(v)$, which are both functions of control effort *v*(*t*) , which, in turn, is a function of time, namely:

1 $$\begin{aligned} \min \limits _{v \in V} (J_b(v)+J_c(v)) \end{aligned}$$

with disease dynamics described by the above SIRW model. The control effort *v*(*t*) is assumed to be continuous over time and is subject to constraints $0 \le v(t) \le v_{\max }$, with $v_{\max }$ being the maximum vaccination rate that can be deployed during the simulation time $0 \le t \le T$.

The total cost of intervention is a nonlinear function of *v*(*t*),

$$\begin{aligned} J_c(v) = \int _0^T [B v^2(t) + C v(t)S(t)]\,\textrm{d}t, \end{aligned}$$

where *B* and *C* are positive constants accounting for the cost of vaccination. We take a simple cost functional to reflect the possibility of nonlinearity in costs; other formats could used depending on the knowledge surrounding costs. Later in our examples, we will illustrate the case where the nonlinearity plays a role and a case where *B* is small enough that the optimal control is approximately bang-bang, meaning the optimal control only takes values at its lower and upper bounds. We note that if $B=0$, then this problem is linear in the controls. In some applications, this could result in possible singular controls as part of the solution, which can occur when the derivative of the Hamiltonian with respect to the control vanishes on a non-empty subinterval of time, requiring further analysis. See the work by Ledzewicz and Schaettler for a simple ${\textit{SIR}}$ model exhibiting singular controls, and see their book for some further background on optimal control problems that are linear in the control (Ledzewicz and Schättler 2011; Schättler and Ledzewicz 2012).

For certain diseases, the infected nature of some individuals, who are able to transmit the pathogen, may not be observed. They could be asymptomatic, and it is possible that those persons might be vaccinated. In such cases, the cost of vaccinations in the objective functional would include a term in the integrand, like $Cv(t)(S(t) +A(t))$, with *A* for the asymptomatic class.

The cost associated with the disease depends upon the specific disease system under study and the available information. It can be represented as

$$\begin{aligned} J_b(v) = \int _0^T A_b I(t)\,\textrm{d}t, \end{aligned}$$

where $A_b$ is a positive scaling coefficient accounting for the aggregated mean monetary value $c_1$ of each day of illness (including drug treatment and loss of productivity) and, in case of fatal disease, also for the value $c_2$ of human lives lost, namely:

$$\begin{aligned} A_b = c_1 + c_2 \delta , \end{aligned}$$

where $\delta I$ is the rate of individuals dying because of the disease. In many examples with $A_b =c_1$, the first term of the objective functional integrates the number of current infected individuals, and we illustrate this frequently used ‘burden’ case in the Appendix.

As an alternative method of computation, the monetary cost of disease only, not accounting for deaths, might be also estimated as a flat rate associated to each infected case and, as such, derived from total disease incidence, i.e., the total number of infected cases during the simulation time, namely:

$$\begin{aligned} J_n(v) = \int _0^T A_n [\beta _I S(t)I(t) + \beta _W S(t)W(t)]\,\textrm{d}t, \end{aligned}$$

with the $\beta$ terms giving the rate of new cases.

The precise form of optimal control is affected by the relative values of the coefficients $A_b, A_n, B, C, c_1$ and $c_2$. Integrating the sum of the rates of the new infections gives us the total number of new cases. The optimal control can be found using the Pontryagin’s Maximum Principle (Pontryagin et al. 1962) as described later in “Appendix A”. We would also note that one can solve for the value function and the resulting optimal control using Hamilton–Jacobi–Bellman partial differential equation approach (with viscosity solution) as in (Laguzet and Turinici 2015; Bardi and Capuzzo-Dolcetta 1997; Piunovskiy and Clancy 2008).

One could include terms in the objective functional at the final time, like *I*(*T*), which would affect the optimal control levels at the final time. There may situations where an infinite time horizon makes sense for particular applications; optimal control problems on infinite horizons are frequently used in economic models (Miclo et al. 2022; Naveed et al. 2019; Kamien and Schwartz 1991).

### Combined Formulations

*Combined Objective Functional with Disease Burden and Cost*

The objective functional for minimizing both disease burden and cost is defined as

2 $$\begin{aligned} \min \limits _{v \in V} \int _0^T [A_bI(t)+Bv^2(t)+Cv(t)S(t) ]\,\textrm{d}t, \end{aligned}$$

where the control set is

$$\begin{aligned} V = \{ v \in L^2(0,T)\,| \, 0 \le v(t) \le v_{\max } \, a.e.\}. \end{aligned}$$

*Combined Objective Functional with New Infections and Cost*

The objective functional for minimizing both the total number of new infections and intervention cost is defined as

3 $$\begin{aligned} \min \limits _{v \in V} \int _0^T [A_n(\beta _I S(t)I(t) + \beta _W S(t)W(t)) +Bv^2(t)+Cv(t)S(t) ]\,\textrm{d}t. \end{aligned}$$

### Alternate Formulation 1: Minimize New Infections under Fixed Budget

Given a fixed budget *G* for the cost of the control interventions, we define the admissible control set (with constraint)

$$\begin{aligned} F_1 = \left\{ v \in V \, | \, \int _0^T (Bv^2(t)+Cv(t)S(t))\,\textrm{d}t \le G \right\} , \end{aligned}$$

where *S*(*t*) is the susceptible compartment of the population corresponding to the control *v*(*t*). The objective functional to minimize the new infections is defined as

4 $$\begin{aligned} \min \limits _{v \in F_1 } J_n(v) = \min \limits _{v \in F_1} \int _0^T A_n[\beta _I S(t)I(t) + \beta _W S(t)W(t) ]\,\textrm{d}t, \end{aligned}$$

subject to the above SIRW model.

To illustrate the process of obtaining the adjoint functions and the optimal control characterization, we use Pontryagin’s Maximum Principle (Pontryagin et al. 1962). To handle the budget constraint, we introduce a fifth state variable, $x_5$, that satisfies

$$\begin{aligned} x_5' = Bv^2 + CvS \end{aligned}$$

with the boundary conditions $x_5(0)=0$ and $x_5(T)=G$. Then, the Hamiltonian using this principle for our optimization is

$$\begin{aligned} H&= A_n \beta _I SI + A_n \beta _W SW + \lambda _1[\mu N -\beta _ISI-\beta _WSW - \mu S -vS] \\&\quad +\lambda _2[\beta _W WS + \beta _I SI - \gamma I - \mu I -\delta I] \\&\quad + \lambda _3 [\gamma I - \mu R +v(t) S] \\&\quad + \lambda _4[\xi ( I - W )]\\&\quad + \lambda _5[Bv^2+CvS ] \end{aligned}$$

with the following adjoint differential equations and boundary conditions

$$\begin{aligned} \lambda _1'&= -[A_n \beta _I I + A_n \beta _W W -\lambda _1 \beta _I I - \lambda _1 \beta _W W - \lambda _1 v + \lambda _2 \beta _W W \\&\quad + \lambda _2 \beta _I I + \lambda _3 v + \lambda _5 C v] \\ \lambda _2'&= -[A_n \beta _I S + \lambda _1 \mu - \lambda _1 \beta _I S + \lambda _2 \beta _I S - \lambda _2 \gamma - \lambda _2 \mu - \lambda _2 \delta + \lambda _3 \gamma + \lambda _4 \xi ]\\ \lambda _3'&= -[\lambda _1 \mu - \lambda _3 \mu ]\\ \lambda _4'&= -[A_n \beta _W S -\lambda _1 \beta _W S + \lambda _2 \beta _W S - \lambda _4 \xi ]\\ \lambda _5'&=0 \\ \lambda _1(T)&= 0, \lambda _2(T)=0, \lambda _3(T)=0, \lambda _4(T)=0. \end{aligned}$$

The adjoint system is formed by $\lambda _i' = - \frac{\partial H}{\partial x_i}$, where $x_i$ represents the *i*th state variable. Note $\lambda _5$ takes no boundary condition since $x_5$ takes two boundary conditions.

We can then characterize the optimal control on the interior of the control set using the optimality condition

$$\begin{aligned} \frac{\partial H}{\partial v} = 2B \lambda _5 v + C \lambda _5 S - \lambda _1 S + \lambda _3 S =0 \text { at } v^* \end{aligned}$$

at the corresponding optimal states, adjoints, and control bounds to yield

$$\begin{aligned} v^*(t) = \min \left( v_{\max }, \max \left( 0, \frac{(\lambda _1(t) - \lambda _3(t)-C \lambda _5 (t))S^*(t)}{2 \lambda _5(t) B}\right) \right) . \end{aligned}$$

### Alternate Formulation 2: Minimize Intervention Costs with Fixed New Infections

Assuming the desired outcome is the cost of number of new infection cases being bounded above by *P*, we define the admissible control set

$$\begin{aligned} F_2 = \left\{ v \in V \, \vert \, \int _0^T A_n[\beta _I S(t)I(t) + \beta _W S(t)W(t)]\,\textrm{d}t \le P \right\} . \end{aligned}$$

The objective functional, defined to minimize the costs of implementing the control, is

5 $$\begin{aligned} \min \limits _{v \in F_2 } J_c(v) = \min \limits _{v \in F_2} \int _0^T [Bv^2(t)+Cv(t)S(t)]\,\textrm{d}t, \end{aligned}$$

subject to the above SIRW model. To handle the constraint on the new cases, we introduce a new state variable, $x_5$ with

$$\begin{aligned} x_5'=A_n[\beta _I S(t)I(t) + \beta _W S(t)W(t)], \;\, x_5(0)=0, \;\, x_5(T)=P \end{aligned}$$

similar to the previous formulation.

Note that $A_n$ is included in (4) and (5) to show the connection between the combined and alternate formulations and allow us to demonstrate how the results of the combined case can be recovered from the alternate cases. However, to achieve the goal of reformulating optimal control problems to avoid the monetization of human health, this constant can be redefined so that the objective functional represents minimizing only the total number of new infections and not the monetary cost associated with these infections. In the numerical illustrations below, we will set $A_n=1$, since the relative sizes of the coefficients determine the optimal control.

## Results

Note that in the results below, we choose a variety of parameters to illustrate various points.

### OC Solutions are Superior to the Best Alternative with Constant Control

Modulating control variables in time may provide a more efficient and cost-effective intervention strategy than the best alternative with a constant control. This point is illustrated by using our example cholera model and a combined objective functional (3) to minimize the cost of interventions plus the cost of the cumulative number of new cases using parameters as given in Table . Our numerical results in this paper use the forward-backward sweep algorithm (Lenhart and Workman 2007). Other algorithms and software programs such as GPOPS and PASA have been developed to handle particular types of optimal control problems (Hager and Zhang 2016; Patterson and Rao 2014).

We first illustrate the advantage of time varying control as compared to a constant control. Note that quadratic coefficient *B* in our objective functional is large enough that our optimal control is not bang-bang; it takes values from its upper bound to lower bounds, connecting in between. The results shown in Fig.  and Table 2 show that the combined cost of intervention and new cases, $J_n(v)+J_c(v)$, is much larger in the case of no control than the cases of optimal or constant control. We choose the constant control to have approximately the same intervention costs as the optimal control. The controlled cases perform equivalently in terms of intervention costs, though with the optimal control case giving better health outcomes. In the optimal control case, the control is time varying, staying at the maximum for approximately 40 days and then decreasing linearly to zero over the next 20 days. A constant vaccination rate $v \equiv 0.032$ leads to approximately the same intervention cost (black line in the plot) but with a higher number of cases. Decreasing the number of deaths by 10% by using our optimal time-varying control seems valuable. Also note that the maximum number of infected individuals occurs at about 30 days at a level of about 3800, but in the uncontrolled case, the number of infected individuals is about 10,000 near the final time and still rising. There is no constant value of vaccination rate for which the combined costs is lower than in the case of the optimal control solution, since all constant controls are admissible controls but the optimal control is not constant with this choice of parameters.

**Fig. 1:**
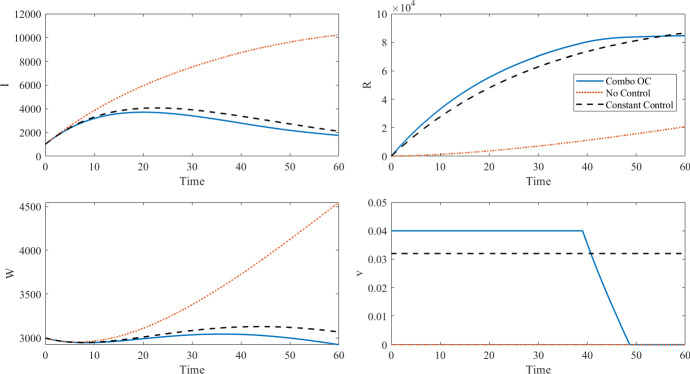
Optimal control and states for the combined objective functional (3) with new infections compared to a constant control with the same cost and the no control case, with parameters as in Table 2

**Table 2:** Results of combined objective functional with time horizon of 60 days corresponding to Fig. 1, using $A_n=1$, $B=8000$, $C=0.0464$, $v_{\max }=0.04$, $\beta _I=2.64e-7$, $\beta _W=1.21e-6$, $\gamma = 0.05$, $\mu =0.0001$, $\xi = 7.56e-3$, $\delta = 0.0005$, $S(0) = 99{,}000$, $I(0)=1000$, $R(0)=0$, and $W(0)=3000$

	No Control	Comb. OC	$v \equiv 0.032$
Disease cost $J_n(v)$	30,219	9337	10,829
Intervention costs $J_c(v)$	0	4090	4079
Total cost $$J_n(v) +J_c(v) $$	30,219	13,427	14,908
Max number of infected	10,219	3712	4072
Total number of deaths	208	85	96
Number of vaccinations	0	76,514	77,325

The $R_0$ for these simulations is 2.9

### Simulations of Alternative Formulations

The optimal control policy is sensitive to how we estimate the monetary cost of the disease, which is notably fraught with uncertainty. For instance, following Castonguay et al. (2020), we can reasonably assume that each sick day entails a productivity loss equal to the daily per-capita Gross Domestic Product (GDP). However, in Africa, the per-capita GDP spans over a 15 fold difference between the richest countries (Gabon, Botsawa, Equatorial Guinea, Mauritius and Seychelles, all above $15,000 per year) and the poorest ones (Burundi, Somalia, Central African Republic, all below $1000/year) (The World Bank 2021). Keeping this in mind, to illustrate the implications of such a wide variation in the per-capita, daily productivity loss, we simulated cholera epidemic dynamics and derived the OC policy with the combined formulation (3) under two alternative assumptions, namely that the unit cost $$A_n$$ of each new case is assumed to be equal to either 1 or 10 in suitable monetary units to measure the average productivity loss while sick. For the results shown in Fig.  and Table , we use the same $$\beta _I$$, $$\beta _W$$, $$\mu $$, $$\xi $$, and $$\delta $$ parameters, initial conditions, and time horizon as in Table 2 and set $$\gamma = 0.25$$, $$B=100$$, $$C=0.1625$$, $$v_{\max }=0.03$$. Due to the change in $$\gamma $$, the $$R_0$$ for these simulations is 0.59. Note that due to the parameters chosen, especially a smaller *B*, the optimal controls in these simulations are approximately bang-bang (the optimal control only takes values at its upper and lower bounds). Also the vaccination campaign starts near the time of the peak of infected population, and thus the optimal controls do not affect that peak but do affect the number of deaths and vaccinations, depending on the size of $$A_n$$.

**Fig. 2:**
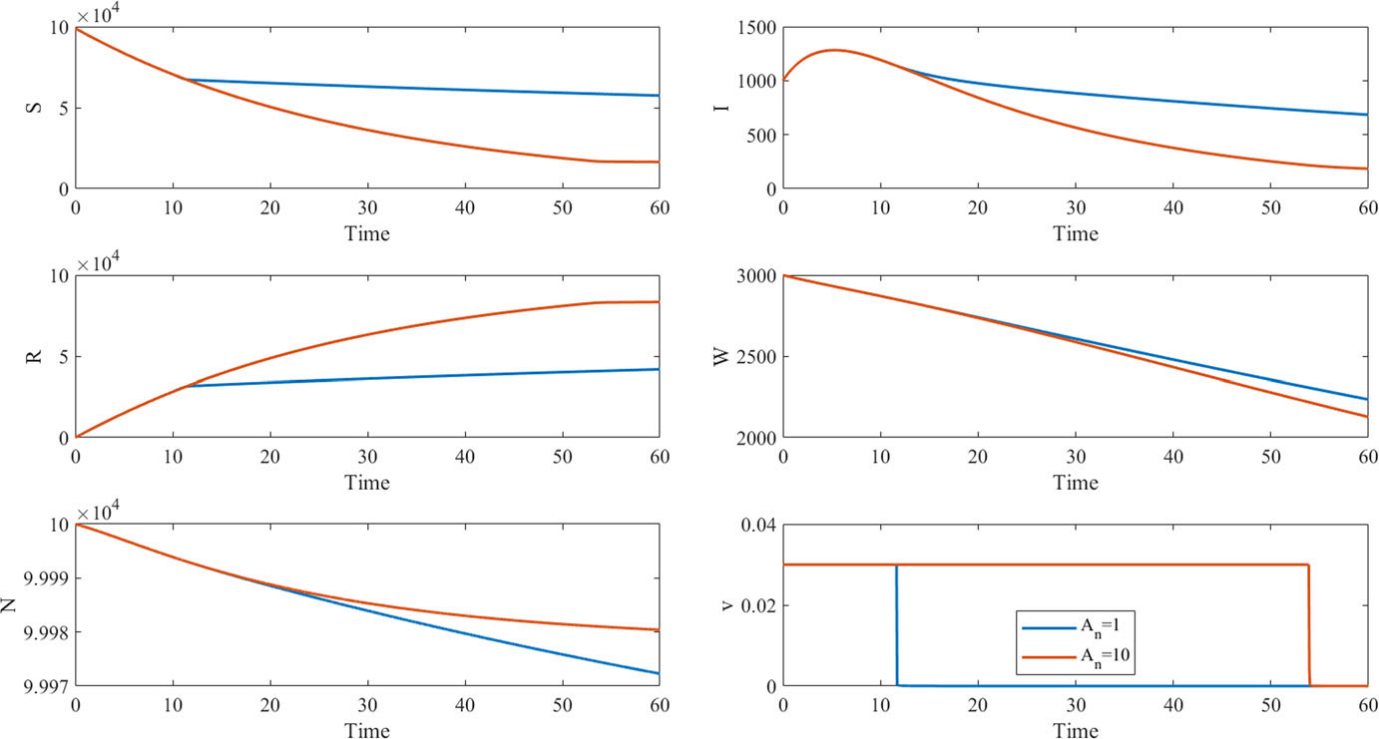
Optimal control and corresponding states for the combined objective functional for different $A_n$ values corresponding to the results in Table 3

Simulations with our cholera model show that, when the unit cost of disease is undervalued by an order of magnitude (when $A_n=1$), the optimal strategy entails 64% less vaccinations, a 71% reduction in the number of days in which vaccines are deployed at the maximum rate, 40% more lives lost, and 51% more cases (Fig. 2/Table 3). Reformulating the OC problem to minimize disease incidence under budget constraints or, in alternative, to minimize intervention costs with a cap on cumulative incidence, allows us to overcome the challenges in estimating the monetary value of the disease, as well as the ethical perils and the uncertainties in the combined formulation.

**Table 3:** Epidemiological values corresponding to Fig. 2 where the percent differences are measured from $A_n=10$ case

	$A_n=10$	$A_n=1$	% difference
Cumulative cases	9011	13,580	+ 51%
Disease cost $J_n(v)$	90,105	13,580	$-$ 85%
Intervention costs $J_c(v)$	12,011	4581	$-$ 62%
Total cost $$J_n(v) +J_c(v) $$	102,116	18,161	$$-$$ 82%
Total number of deaths	20	28	+ 40%
Number of vaccinations	73,654	26,634	$-$ 64%
Time spent at $v_{\max }$	55	16	$-$ 71%

Do the alternative formulations systematically lead to different control policies than the OC policy derived by solving the combined formulation? Our analysis shows that, in the special (and unrealistic) case of perfect information—i.e., when the monetary cost of the disease can be derived with sufficient precision—budget or epidemiological constraints can be set so that the OC policy resulting from the alternative formulations is the same as the one computed with the combined formulation (3). Specifically, we solved the OC problem for the first alternative formulation (i.e., minimize new infections under a budget constraint (4)) by setting the budget constraint to the intervention costs resulting from solving the combined OC problem (3). As shown in Table  and Fig. , the OC policy and health outcomes (such as the maximum number of infected individuals, the number of deaths, and the number of vaccinations) are the same for the combined and the alternative formulations since the intervention cost budget is the same for both. We see the same for the second alternative formulation (i.e., minimize intervention costs to achieve a desired epidemiological outcome (5)) when the epidemiological outcome is constrained to the value derived by solving the combined OC problem (see Table  and Fig. ).

**Fig. 3:**
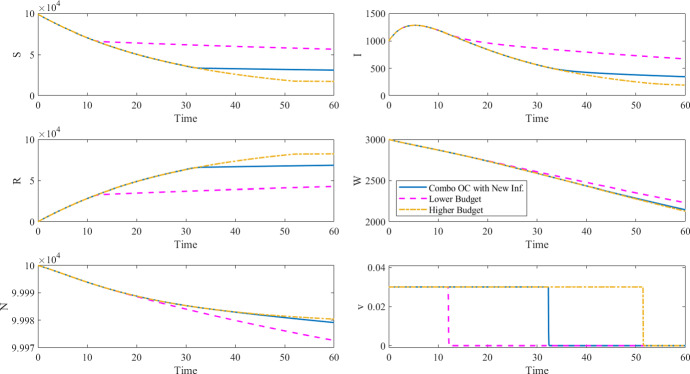
Optimal control and corresponding states for the combined objective functional with new infections (3) and the objective functional with new infections under a budget constraint (4), corresponding to the results in Tables 4 and 6

**Fig. 4:**
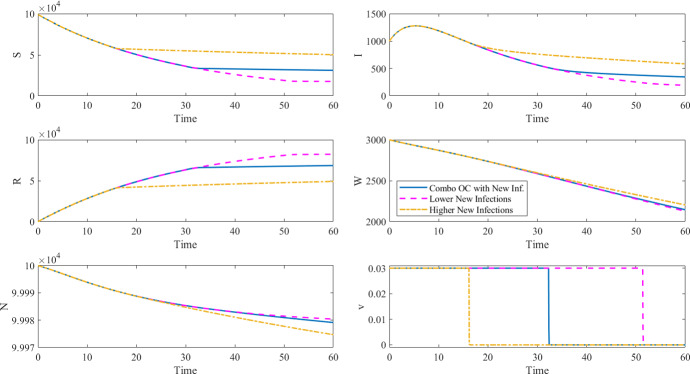
Optimal control and corresponding states for the combined objective functional with new infections (3) and the objective functional minimizing cost under an epidemiological constraint (5), corresponding to the results in Tables 5 and 7

The simulations shown in Fig. 3 (corresponding to Tables 4 and ) and Fig. 4 (corresponding to Tables 5 and ) were performed using the same $\beta _I$, $\beta _W$, $\mu$, $\xi$, and $\delta$ parameters, initial conditions, and time horizon as in Table 2 and setting $\gamma = 0.25$, $A_n=1$, $B=100$, $C=0.0813$, $v_{\max }=0.03$.

**Table 4:** Results of the objective functional with new infections under a budget constraint (4) compared to the case of no control and the combined objective functional with new infections (3), corresponding to the results in Fig. 3

	No control	Comb. OC	Alt. OC
Intervention costs $J_c(v)$	0	4746	$4746 ^{*}$
Disease cost $J_n(v)$	19,080	9814	–
Total costs $J_n(v)+J_c(v)$	19,080	$14,559 ^{\dagger }$	–
Cumulative cases	19,080	9814	$9814 ^{\dagger }$
Max number of infected	1459	1282	1282
Number of deaths	38	21	21
Number of vaccinations	0	57,501	57,501

$ values are hypothetical and the $ symbol is used to clearly tease apart monetary values from health outcomes. The $$^{*}$$ symbol for the alternative OC formulation indicates that the OC problem was solved by setting the intervention cost as a constraint. The $$^{\dagger }$$ symbol indicates what value was minimized in the OC problem

Then what advantage do the alternative formulations provide? As commented above, in the majority of cases, the per-capita cost of disease is not known and cannot be estimated with any degree of confidence, especially in the case of fatal or debilitating chronic diseases. Therefore, it is more meaningful and less debatable from the perspective of health equity to frame the optimization problem by explicitly acknowledging the fundamental reality any decision maker has to face. That is, to operate in conditions of limited budget, and/or, to have ethical, political, practical or humanitarian reasons to achieve a desired public health outcome. The alternative formulations allow us to incorporate these considerations much more straightforwardly than the combined formulation does. For instance, in the last two columns of Table 6 we report the breakdown for costs and epidemiological outcomes associated with a 50% budget decrease or a 25% increase, respectively, compared to the intervention cost found in the combined OC solution. The last two columns of Table 7 report the breakdown of the same information when the epidemiological target is 8% lower or 25% higher than the baseline number of new infections (as derived from the combined OC solution).

Note that, in the simulation results shown in Tables 6 and 7, the maximum number of new infections does not change between the different budget and epidemiological constraints. This is because the peak of infections in those simulations occurs early in the simulation and the optimal controls in all of the cases shown are at the maximum control value from the beginning of the simulation until after the peak of infection. Therefore, in these cases, the maximum number of infected is not reduced by increasing budget or setting a more ambitious epidemiological goal. To reduce the maximum number of infected individuals in these simulations, an increase in the maximum vaccination rate is required. An example of a simulation with a different maximum number of infected individuals can be seen in Fig. 1, where a constant control is applied at a lower maximum vaccination rate than the combined optimal control case. Therefore the constant control case displays an increased maximum number of infected individuals.

**Table 5:** Results of the objective functional with intervention costs and an epidemiological constraint (5) compared to the no control case and the combined objective functional with new infections (3), corresponding to the results in Fig. 4

	No Control	Comb. OC	Alt. OC
Intervention costs $J_c(v)$	0	4746	$4746^{\dagger }$
Disease cost $J_n(v)$	19,080	9814	–
Total costs $$J_n(v) +J_c(v) $$	19,080	$$ 14,559 ^{\dagger } $$	–
Cumulative cases	19,080	9814	$9814^{*}$
Max number of infected	1459	1282	1282
Number of deaths	38	21	21
Number of vaccinations	0	57,501	57,501

The $^{*}$ symbol for the alternative OC formulation indicates that the OC problem was solved by setting the cumulative cases as a constraint. The $^{\dagger }$ symbol indicates what value was minimized in the OC problem

**Table 6:** Results of the combined objective functional with new infections (3) and the objective functional with new infections and a fixed budget (4) corresponding to the results in Fig. 3

	Alt. OC	Lower budget	Higher budget
Intervention costs $$J_c(v)$$	$$4746 ^{*}$$	$$2373 ^{*}$$ ($$-$$ 50%)	$$ 5934 ^{*}$$ (+ 25%)
Cumulative cases	$9814 ^{\dagger }$	$13,416 ^{\dagger }$ (+ 37%)	$9028 ^{\dagger }$ ($-$ 8%)
Max number of infected	1282	1282 (0%)	1282 (0%)
Number of deaths	21	27 (+ 29%)	20 ($-$ 5%)
Number of vaccinations	57,501	30,624 ($-$ 47%)	72,653 (+ 26%)

The $^{*}$ symbol for the alternative OC formulation indicates that the OC problem was solved by setting the intervention cost as a constraint. The $^{\dagger }$ symbol indicates what value was minimized in the OC problem

**Table 7:** Results of the combined objective functional (3) and the objective functional with intervention costs and an epidemiological goal (5), corresponding to the results in Fig. 4

	Alt. OC	Lower New Inf	Higher New Inf
Intervention costs $J_c(v)$	$4746 ^{\dagger }$	$5921 ^{\dagger }$ (+ 25%)	$2983 ^{\dagger }$ ($-$ 37%)
Cumulative cases	$$ 9814^{*}$$	$$9030 ^{*} $$ ($$-$$ 8%)	$$ 12,275^{*} $$ (+ 25%)
Max number of infected	1282	1282 (0%)	1282 (0%)
Number of deaths	21	20 ($-$ 5%)	25 (+ 20%)
Number of vaccinations	57,501	72,507 (+ 26%)	37,846 (34%)

The $^{*}$ symbol for the alternative OC formulation indicates that the OC problem was solved by setting the cumulative cases as a constraint. The $^{\dagger }$ symbol indicates what value was minimized in the OC problem

**Fig. 5:**
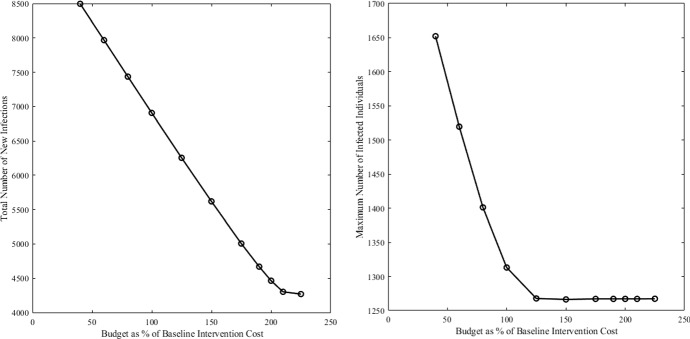
Left: Total number of new infections as a function of the budget constraint for Alternative Formulation 1 (4). Right: Maximum number of infected individuals *I* over the time interval as a function of the budget constraint for Alternate Formulation 1 (4). The budget constraint is set as a percentage of the total intervention cost from the baseline case (the combined OC formulation (3)). These simulations were performed with $A_n=1$, $B=600$, and $C=1$ and using $\beta _I=2.64e-7$, $\beta _W=1.21e-6$, $\gamma = 0.25$, $\mu =0.0001$, $\xi = 7.56e-3$, $\delta = 0.0005$, $S(0) = 99{,}000$, $I(0)=1000$, $R(0)=0$, $W(0)=3000$, and $v_{\max }=0.08$

A sensitivity analysis can be conducted using alternative formulation 1 (minimizing new infections under a budget constraint (4)) by systematically exploring changes in health outcomes of interest over a wide range of budget constraints (see Fig. ). Our analysis shows that the relationship between budget and health outcomes are, for this model, approximately linear until one reaches the budget that uses the maximum vaccination rate over the entire simulation time. The analysis reveals that above this level, what is limiting is not vaccination budget but, rather, vaccination capacity (for instance, the number of vaccination centers). Therefore, above this limit, further health benefits could be achieved by a structural improvement of the vaccination system.

For alternative formulation 2 (minimizing intervention cost to achieve a desired epidemiological outcome (5)), the sensitivity analysis shows that the total intervention cost linearly increases/decreases, respectively, with more ambitious public health targets (percentage reduction in new cases with respect to the no control case—Fig. ). The maximum value of new daily infections drops linearly with more ambitious epidemiological outcome but levels off, in this case, when the required reduction in the cumulative number of cases exceed ca. 30% of that at baseline (no intervention).

**Fig. 6:**
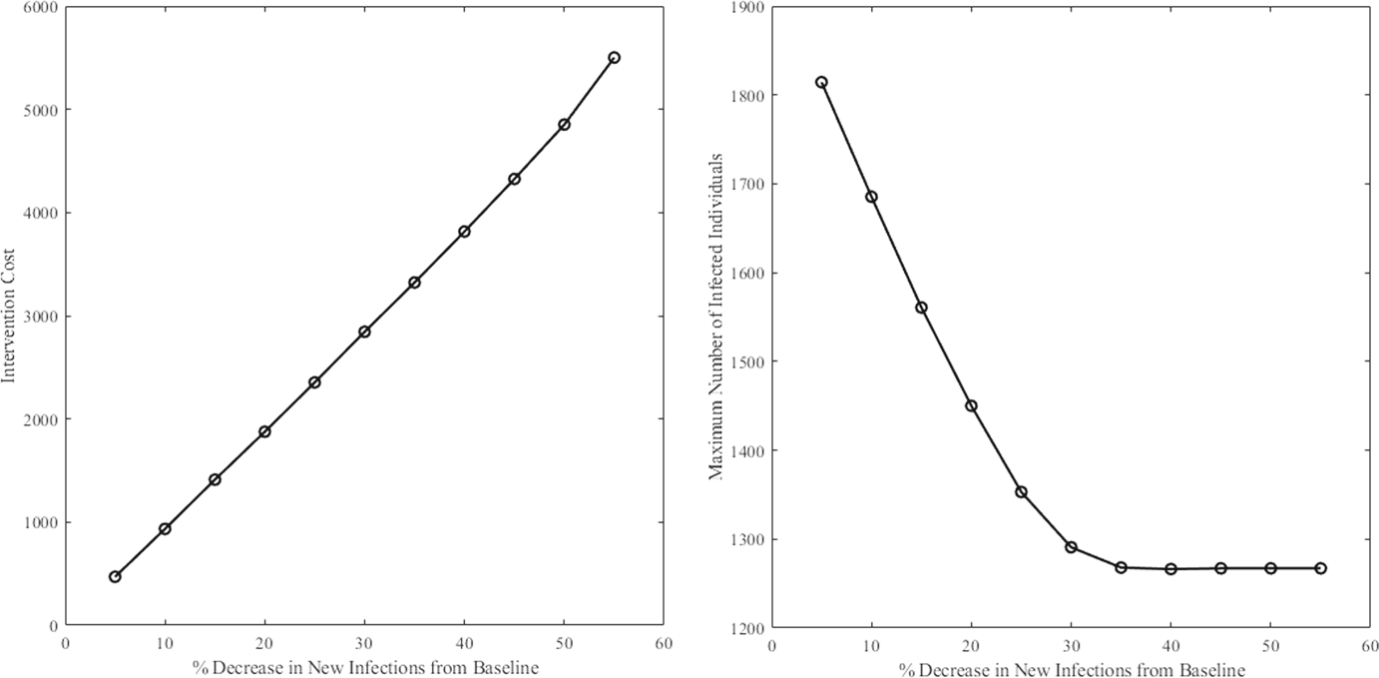
Left: Intervention cost as a function of the epidemiological constraint for Alternative Formulation 2 (5). Right: Maximum number of infected individuals *I* over the time interval as a function of the epidemiological constraint for Alternate Formulation 2 (5). The epidemiological constraint is set as a percent reduction in the number of new infections from the baseline case (the case with no control). These simulations were performed with $A_n=1$, $B=600$, and $C=1$ and using $\beta _I=2.64e-7$, $\beta _W=1.21e-6$, $\gamma = 0.25$, $\mu =0.0001$, $\xi = 7.56e-3$, $\delta = 0.0005$, $S(0) = 99{,}000$, $I(0)=1000$, $R(0)=0$, $W(0)=3000$, and $v_{\max }=0.08$

Simulations related to the optimal control formulations minimizing disease burden instead of new infections (2) can be found in “Appendix B”.

## Discussion and Conclusions

Using a cholera model as a prototypical example of environmentally related infectious disease, we have illustrated the benefit of using time-varying controls to achieve the goals and incorporate the constraints of disease management. We have proposed valuable alternatives to the frequently-used objective functional (the minimization of the sum of the total number of new cases (or total disease burden) and the cost of management actions) and showed the advantages of these alternatives in terms of outcomes (in costs, new cases and/or burden levels). Depending on the situation, one should consider minimizing the number of new cases given a fixed intervention budget (4) or minimizing the intervention costs given a fixed epidemiological constraints (such as keeping the total number of new cases under a fixed level (5)). These formulations can be valuable to apply to models of other diseases, such as schistosomiasis (Sokolow et al. 2015), West Nile virus (Abdelrazec et al. 2016), and Zika virus (Valega-Mackenzie and Ríos-Soto 2018; Miyaoka et al. 2019). We also note that other types of budget constraints, like time-varying upper bounds on costs or subsidies, may be interesting to consider; the budget for each year could be different and could have the option of rolling over each year (Drohan et al. 2021; Rowthorn et al. 2009). If the values of specific epidemiological variables such as the fraction of the population with acquired or vaccine-induced immunity near the end of reference time horizon are important, it is possible to reformulate the objective functional to explicitly account for those variables (with terms at the final time in the objective functional).

We analyzed changes in health outcomes of interest over a range of budget constraints. For the specific model and the parameterization in this paper, this relationship between budget and health outcomes was found to be approximately linear until one reaches the budget level that allows the maximum vaccination rate to be used over the entire simulation time. The upper bound on the vaccination rate may be linked to the capacity of the health-care infrastructure (that is, the amount of vaccinations that can be completed per unit time given the current delivery system with a fixed number of workers). Similar results were obtained for changes in intervention costs over a range of fixed health outcomes. Overall, our research outlines the power of reformulating combined optimal control problems and using health outcomes measured in metrics with which public health decision makers are more familiar.

In some of our results, the differences in vaccination effort and epidemiological outcome merely reflect differences in the economic strengths of nations, and have little to do with the much less tangible value of the quality of life lost due to infection. This problem can be partially addressed by conducting a sensitivity analysis of how the OC solution changes as a function of the unit cost of disease, if such costs are known (see one such analysis in Bolzoni et al. 2014).

While the proposed reformulation of the optimization functional addresses some problems of the combined formulation and allows us to avoid the practical and ethical challenges of monetizing human health, it does not solve other problems inherent in the evaluation of health outcome and equity issues. For instance, Weintraub and Cohen (2009) have clearly outlined several of these limitations for the cost-effectiveness analysis that apply also to optimal control problems in general and, specifically, to the newly proposed formulations. Though monetizing the value of human health comes with uncertainties, estimating intervention costs and translating those costs into model parameters also present challenges. In addition, for practical and computational reasons, mechanistic models tend to simplify the complexity of real-world social systems, and optimal control theory cannot overcome the limitations deriving from oversimplified models that lack relevant details (such as spatial structure or social interaction) or are not properly parameterized because of a lack of the necessary data.

Without dismissing the power of OC applications, we are aware that, during an actual epidemic outbreak and in practical terms, simplified protocols with constant intervention rates might work equally well, if not better, than protocols that require continuous adjustment of the intervention effort. This may be especially true when it is difficult to monitor the health state of the population, testing capacity is low, or there are delays in reporting infected cases in remote, rural areas and/or in countries with limited health care and reporting systems. An optimal control can frequently be adjusted (every few months or so) to give a close approximate control that is more feasible to implement (Morris et al. 2021; Demir and Lenhart 2020).

OC applications to the control of communicable diseases often remains so technical that no actual use of them becomes realized as a guideline for action. While some barriers due to the inherent complexity of OC theory cannot be overcome, there is an untapped potential that should be leveraged to inform the decision making process on pressing public health problems. In spite of the limitations listed above, we believe that the scientific community proficient in optimal control theory could easily formulate optimization problems for the control of communicable diseases with our proposed approach. It would reflect more closely the framework in which public health decisions are usually taken in the full spirit of contemporary user-inspired research, as argued in National Research Council (2008) and Stoles (1997).

## Figures and Tables

**Fig. 7:**
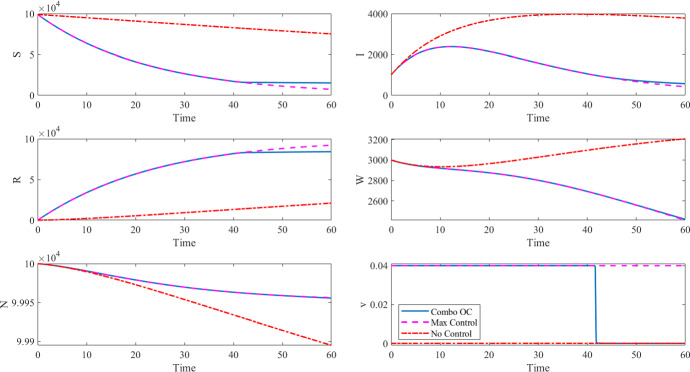
Optimal control and corresponding states for the combined objective functional with disease burden compared to the no control case and a constant control with the above parameters, corresponding to the results in Table 8

**Fig. 8:**
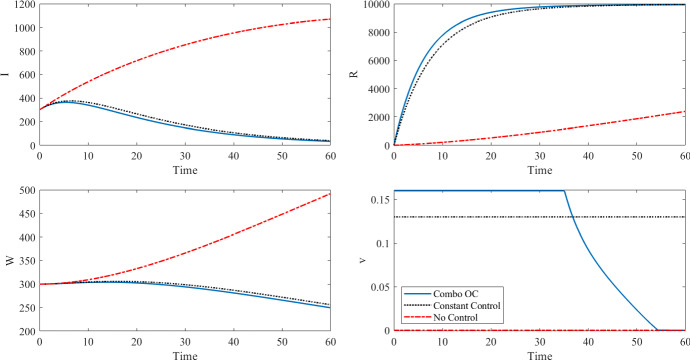
Optimal control and states for the combined objective functional with burden compared to a constant control with the same intervention cost and the no control case, corresponding to results in Table 9

## Data Availability

There are no associated data with this manuscript.

## Appendix A: Hamiltonians, Adjoints, and Optimal Control Characterizations

### Appendix A.1: Combined Objective Functional with Disease Burden and Cost

The objective functional combining disease burden and intervention cost is formulated as

$$\begin{aligned} \min \limits _{v \in V} \int _0^T [A_bI(t)+Bv^2(t)+Cv(t)S(t) ]\,\textrm{d}t, \end{aligned}$$

where the control set is

$$\begin{aligned} V = \{ v \in L^2(0,T) \, | \, 0 \le v(t) \le v_{\max } \; a.e.\}. \end{aligned}$$

The Hamiltonian is then

$$\begin{aligned} H&= A_bI+Bv^2+CvS + \lambda _1[\mu N -\beta _ISI-\beta _WSW - \mu S -vS] \\&\quad +\lambda _2[\beta _W WS + \beta _I SI - \gamma I - \mu I -\delta I] \\&\quad + \lambda _3 [\gamma I - \mu R +v(t) S] \\&\quad + \lambda _4[\xi ( I - W )] \end{aligned}$$

with the following adjoints and boundary conditions:

$$\begin{aligned} \lambda _1'&= -[Cv -\lambda _1 \beta _I I - \lambda _1 \beta _W W - \lambda _1 v + \lambda _2 \beta _W W + \lambda _2 \beta _I I + \lambda _3 v] \\ \lambda _2'&= -[A_b + \lambda _1 \mu - \lambda _1 \beta _I S + \lambda _2 \beta _I S - \lambda _2 \gamma - \lambda _2 \mu - \lambda _2 \delta + \lambda _3 \gamma + \lambda _4 \xi ]\\ \lambda _3'&= -[\lambda _1 \mu - \lambda _3 \mu ]\\ \lambda _4'&= -[-\lambda _1 \beta _W S + \lambda _2 \beta _W S - \lambda _4 \xi ]\\ \lambda _1(T)&= 0, \lambda _2(T)=0, \lambda _3(T)=0, \lambda _4(T)=0. \end{aligned}$$

We can then characterize the optimal control, using this optimality condition, on the interior of the control set.

$$\begin{aligned} \frac{\partial H}{\partial v} = 2Bv + CS - \lambda _1 S + \lambda _3 S = 0 \text { at } v^*. \end{aligned}$$

Note $\frac{\partial ^2 H}{\partial v ^2} = 2B>0$, since $B>0$. Therefore, using the control bounds gives

$$\begin{aligned} v^* = \min \left( v_{\max }, \max \left( 0, \frac{(\lambda _1 - \lambda _3-C)S}{2B}\right) \right) . \end{aligned}$$

### Appendix A.2: Combined Objective Functional with New Infections and Cost

The objective functional combining new infections and intervention costs is formulated as

$$\begin{aligned} \min \limits _{v \in V} \int _0^T [A_n(\beta _I S(t)I(t) + \beta _W S(t)W(t)) +Bv^2(t)+Cv(t)S(t) ],\,\textrm{d}t \end{aligned}$$

where the control set *V* is the same as above.

The Hamiltonian is then

$$\begin{aligned} H&= A_n(\beta _I SI + \beta _W SW) +Bv^2+CvS + \lambda _1[\mu N -\beta _ISI-\beta _WSW - \mu S -vS] \\&\quad +\lambda _2[\beta _W WS + \beta _I SI - \gamma I - \mu I -\delta I] \\&\quad + \lambda _3 [\gamma I - \mu R +v(t) S] \\&\quad + \lambda _4[\xi ( I - W )] \end{aligned}$$

with the following adjoints and boundary conditions:

$$\begin{aligned} \lambda _1'&= -[A_n\beta _I I + A_n\beta _W W + Cv -\lambda _1 \beta _I I - \lambda _1 \beta _W W - \lambda _1 v \\&\quad + \lambda _2 \beta _W W + \lambda _2 \beta _I I + \lambda _3 v] \\ \lambda _2'&= -[A_n\beta _I S + \lambda _1 \mu - \lambda _1 \beta _I S + \lambda _2 \beta _I S - \lambda _2 \gamma - \lambda _2 \mu - \lambda _2 \delta + \lambda _3 \gamma + \lambda _4 \xi ]\\ \lambda _3'&= -[\lambda _1 \mu - \lambda _3 \mu ]\\ \lambda _4'&= -[A_n\beta _W S -\lambda _1 \beta _W S + \lambda _2 \beta _W S - \lambda _4 \xi ]\\ \lambda _1(T)&= 0, \lambda _2(T)=0, \lambda _3(T)=0, \lambda _4(T)=0. \end{aligned}$$

We can then characterize the optimal control, using this optimality condition, on the interior of the control set.

$$\begin{aligned} \frac{\partial H}{\partial v} = 2Bv + CS - \lambda _1 S + \lambda _3 S = 0 \text { at } v^*. \end{aligned}$$

Note $\frac{\partial ^2 H}{\partial v ^2} = 2B>0$, since $B>0$. Therefore, we find that

$$\begin{aligned} v^* = \min \left( v_{\max }, \max \left( 0, \frac{(\lambda _1 - \lambda _3-C)S}{2B}\right) \right) . \end{aligned}$$

### Appendix A.3: Minimize Disease Burden under Fixed Budget

Given a fixed budget *G*, the cost of the control interventions, we define the control sets:

$$\begin{aligned} V = \{ v \in L^2(0,T) \, | \, 0 \le v(t) \le v_{\max } \; a.e.\} \end{aligned}$$

and

$$\begin{aligned} F_1 = \left\{ v \in V \; | \; \int _0^T (Bv^2(t)+Cv(t)S(t))\,\textrm{d}t \le G \right\} , \end{aligned}$$

where *S* is the susceptible compartment of the population corresponding to the control *v*. The alternate objective functional to minimize the disease burden is defined as

$$\begin{aligned} \min \limits _{v \in F_1} \int _0^T A_b I(t)\,\textrm{d}t. \end{aligned}$$

To show the process of obtaining the adjoint functions and the optimal control characterization, we use Pontryagin’s Maximum Principle. To handle the budget constraint, we introduce a fifth state variable, $x_5$, that satisfies

$$\begin{aligned} x_5' = Bv^2 + CvS \end{aligned}$$

with the boundary conditions $x_5(0)=0$ and $x_5(T)=G$.

The Hamiltonian is

$$\begin{aligned} H&= A_bI + \lambda _1[\mu N -\beta _ISI-\beta _WSW - \mu S -vS] \\&\quad +\lambda _2[\beta _W WS + \beta _I SI - \gamma I - \mu I -\delta I] \\&\quad + \lambda _3 [\gamma I - \mu R +v S] \\&\quad + \lambda _4[\xi ( I - W )] \\&\quad + \lambda _5[Bv^2+CvS] \end{aligned}$$

with the following adjoint differential equations and boundary conditions

$$\begin{aligned} \lambda _1'&= -[-\lambda _1 \beta _I I - \lambda _1 \beta _W W - \lambda _1 v + \lambda _2 \beta _W W + \lambda _2 \beta _I I + \lambda _3 v + \lambda _5 C v] \\ \lambda _2'&= -[A_b + \lambda _1 \mu - \lambda _1 \beta _I S + \lambda _2 \beta _I S - \lambda _2 \gamma - \lambda _2 \mu - \lambda _2 \delta + \lambda _3 \gamma + \lambda _4 \xi ]\\ \lambda _3'&= -[\lambda _1 \mu - \lambda _3 \mu ]\\ \lambda _4'&= -[-\lambda _1 \beta _W S + \lambda _2 \beta _W S - \lambda _4 \xi ]\\ \lambda _5'&=0 \\ \lambda _1(T)&= 0, \lambda _2(T)=0, \lambda _3(T)=0, \lambda _4(T)=0. \end{aligned}$$

Note $\lambda _5$ takes no boundary condition since $x_5$ takes two boundary conditions.

We can then characterize the optimal control, using this optimality condition, on the interior of the control set,

$$\begin{aligned} \frac{\partial H}{\partial v} = 2B \lambda _5 v + C \lambda _5 S - \lambda _1 S + \lambda _3 S =0 \text { at } v^*, \end{aligned}$$

and then obtain

$$\begin{aligned} v^*(t) = \min \left( v_{\max }, \max \left( 0, \frac{(\lambda _1(t) - \lambda _3(t)-C \lambda _5 (t))S(t)}{2 \lambda _5(t) B}\right) \right) . \end{aligned}$$

### Appendix A.4: Minimize Intervention Costs with Fixed New Infections

Given an upper bound *P* on the number of new infections, we define the control set $F_2$ as

$$\begin{aligned} F_2 = \left\{ v \in V \, \vert \, \int _0^T A_n[\beta _I S(t)I(t) + \beta _W S(t)W(t)]\,\textrm{d}t \le P \right\} , \end{aligned}$$

where *S* is the susceptible compartment, *I* the infected compartment, and *W* the environmental contamination compartment of the population corresponding to the control *v*.

The alternative objective functional minimizing cost is defined as

$$\begin{aligned} \min \limits _{v \in F_2} \int _0^T [Bv^2(t)+Cv(t)S(t)]\,\textrm{d}t. \end{aligned}$$

To handle the constraint on the new cases, we introduce a new state variable, $x_5$ with

$$\begin{aligned} x_5'=A_n[\beta _I S(t)I(t) + \beta _W S(t)W(t)], \; x_5(0)=0, \; x_5(T)=P. \end{aligned}$$

The Hamiltonian is then

$$\begin{aligned} H&= Bv^2+CvS + \lambda _1[\mu N -\beta _ISI-\beta _WSW - \mu S -vS] \\&\quad +\lambda _2[\beta _W WS + \beta _I SI - \gamma I - \mu I -\delta I] \\&\quad + \lambda _3 [\gamma I - \mu R +v(t) S] \\&\quad + \lambda _4[\xi ( I - W )] + \lambda _5 [A_n\beta _I SI + A_n \beta _W SW] \end{aligned}$$

with the following adjoints and tranversality conditions:

$$\begin{aligned} \lambda _1'&= -[Cv -\lambda _1 \beta _I I - \lambda _1 \beta _W W - \lambda _1 v + \lambda _2 \beta _W W + \lambda _2 \beta _I I \\&\quad + \lambda _3 v +A_n \lambda _5 \beta _I I + A_n \lambda _5 \beta _W W ] \\ \lambda _2'&= -[ \lambda _1 \mu - \lambda _1 \beta _I S + \lambda _2 \beta _I S - \lambda _2 \gamma - \lambda _2 \mu - \lambda _2 \delta + \lambda _3 \gamma + \lambda _4 \xi + A_n \lambda _5 \beta _I S ]\\ \lambda _3'&= -[\lambda _1 \mu - \lambda _3 \mu ]\\ \lambda _4'&= -[-\lambda _1 \beta _W S + \lambda _2 \beta _W S - \lambda _4 \xi + A_n \lambda _5 \beta _W S]\\ \lambda _5'&= 0 \\ \lambda _1(T)&= 0, \lambda _2(T)=0, \lambda _3(T)=0, \lambda _4(T)=0. \end{aligned}$$

Note that $\lambda _5$ takes no boundary condition because $x_5$ takes two boundary conditions, $x_5(0)=0$ and $x_5(T)=P$.

We can then characterize the optimal control, using this optimality condition, on the interior of the control set.

$$\begin{aligned} \frac{\partial H}{\partial v} = 2Bv + CS - \lambda _1 S + \lambda _3 S = 0 \text { at } v^* \end{aligned}$$

Note that $\frac{\partial ^2 H}{\partial v ^2} = 2B>0$, since $B>0$. Therefore, we find that

$$\begin{aligned} v^* (t) = \min \left( v_{\max }, \max \left( 0, \frac{(\lambda _1 (t) - \lambda _3(t)-C)S(t)}{2B}\right) \right) . \end{aligned}$$

### Appendix A.5: Minimize New Infections under Fixed Budget

Given a budget constraint *G*, we use the same control set as “Appendix A.3”,

$$\begin{aligned} F_1 = \left\{ v \in V \, | \, \int _0^T (Bv^2(t)+Cv(t)S(t))\,\textrm{d}t \le G \right\} . \end{aligned}$$

The alternative objective functional minimizing the new infections is then defined as

$$\begin{aligned} \min \limits _{v \in F_1} \int _0^T A_n[\beta _I S(t)I(t) + \beta _W S(t)W(t) ]\,\textrm{d}t. \end{aligned}$$

To handle the budget constraint, we introduce a fifth state variable, $x_5$, defined by

$$\begin{aligned} x_5' = Bv^2 + CvS \end{aligned}$$

with the boundary conditions $x_5(0)=0$ and $x_5(T)=G$.

The Hamiltonian is then

$$\begin{aligned} H&= A_n \beta _I SI + A_n \beta _W SW + \lambda _1[\mu N -\beta _ISI-\beta _WSW - \mu S -vS] \\&\quad +\lambda _2[\beta _W WS + \beta _I SI - \gamma I - \mu I -\delta I] \\&\quad + \lambda _3 [\gamma I - \mu R +v(t) S] \\&\quad + \lambda _4[\xi ( I - W )]\\&\quad + \lambda _5[Bv^2+CvS ] \end{aligned}$$

with the following adjoints and boundary conditions:

$$\begin{aligned} \lambda _1'&= -[A_n \beta _I I + A_n \beta _W W -\lambda _1 \beta _I I - \lambda _1 \beta _W W - \lambda _1 v + \lambda _2 \beta _W W\\&\quad + \lambda _2 \beta _I I + \lambda _3 v + \lambda _5 C v] \\ \lambda _2'&= -[A_n \beta _I S + \lambda _1 \mu - \lambda _1 \beta _I S + \lambda _2 \beta _I S - \lambda _2 \gamma - \lambda _2 \mu - \lambda _2 \delta + \lambda _3 \gamma + \lambda _4 \xi ]\\ \lambda _3'&= -[\lambda _1 \mu - \lambda _3 \mu ]\\ \lambda _4'&= -[A_n \beta _W S -\lambda _1 \beta _W S + \lambda _2 \beta _W S - \lambda _4 \xi ]\\ \lambda _5'&=0 \\ \lambda _1(T)&= 0, \lambda _2(T)=0, \lambda _3(T)=0, \lambda _4(T)=0. \end{aligned}$$

Note $\lambda _5$ takes no boundary condition since $x_5$ takes two boundary conditions.

We can then characterize the optimal control, using this optimality condition, on the interior of the control set.

$$\begin{aligned} \frac{\partial H}{\partial v} = 2B \lambda _5 v + C \lambda _5 S - \lambda _1 S + \lambda _3 S =0 \text { at } v^* \end{aligned}$$

Note that $\frac{\partial ^2 H}{\partial v ^2} = 2B \lambda _5>0$ if $\lambda _5>0$.

Therefore, we find that

$$\begin{aligned} v^* = \min \left( v_{\max }, \max \left( 0, \frac{(\lambda _1 - \lambda _3-C \lambda _5)S}{2 \lambda _5 B}\right) \right) . \end{aligned}$$

## Appendix B: Combined OC: Minimize the Sum of Disease Burden and the Costs of Interventions

Minimizing the cost of interventions and the integral of the infected compartment, scaled appropriately to represent the cost of disease burden, is commonly seen as the goal in management of disease models. The case of a combined objective functional with burden (Eq. 2) is illustrated here in Fig.  with model parameters listed in the caption of Table . In that table, one can see the positive effects of implementing the optimal control on the disease are striking. Since the number of susceptible individuals has become low about half way through the time interval, the constant control (set at the maximum control value, 0.04) has similar effects to the optimal control resulting from the combined objective functional (1% reduction in disease burden), but has a slightly higher intervention cost compared to the optimal control (11% increase). These simulations show that considering a time varying control allows for a better result (i.e. a smaller objective functional) than a constant control at the maximum will allow, by 1.4% in this case.

**Table 8:** Results of the combined objective functional with disease burden compared to the no control case and a constant control of 0.16 for a time horizon of 60 days and with $\beta _I=2.64e-7$, $\beta _W=1.21e-7$, $\gamma = 0.1$ and $A_b=1$, $B=1000$, and $C=0.325$. The initial population was set to $S=99{,}000$, $I=1000$, $R=0$, and $W=3000$

	No Control	Comb. OC with Burden	Constant control
Intervention costs $J_c(v)$	0	24,648	27,314
Disease burden cost $J_b(v)$	210,159	88,907	87,802
Total cost $J_b(v)+J_c(v)$	210,159	113,555	115,116
% difference in total objective functional from constant control	83	$-$ 1.4	–
Max number of infected	3975	2383	2383
Number of deaths	105	44	43

The $R_0$ for this simulation is 1.47

Using the parameters in Table  and a combined objective functional with burden (Eq. 2), we compare the optimal control to a constant control with same cost and to the no control case. The plots shown in Fig.  do not show much difference in the dynamics of the compartments when comparing the optimal control case and a constant control of 0.13. The two controls result in similar intervention costs but have 10% difference in the overall combined cost.

**Table 9:** Results of combined objective functional with disease burden with time horizon of 60 days and $A_b=1$, $B=40$, $C=0.0464$, $v_{\max }=0.16$, $\beta _I=2.64e-6$, $\beta _W=1.21e-5$, $\gamma = 0.05$, $S(0) = 9700$, $I(0)=300$, $R(0)=0$, and $W(0)=300$

	No Control	Comb. OC	$v \equiv 0.13$
Disease burden cost $J_b(v)$	47,933	10,576	11,658
Intervention costs $J_c(v)$	0	479	477
Total cost $$J_b(v) +J_c(v) $$	47,933	11,055	12,135
Max number of infected	1069	363	376
Number of deaths	24	5	6
Number of vaccinations	0	9460	9422

The $R_0$ for this simulation is 2.9
